# Applying ‘timeliness’ to the screening and prevention of TB in household contacts of pulmonary TB patients

**DOI:** 10.5588/ijtldopen.23.0615

**Published:** 2024-02-01

**Authors:** A. D. Harries, D. Nair, P. Thekkur, R. Ananthakrishnan, R. Thiagesan, J. M. Chakaya, I. Mbithi, B. Jamil, R. Fatima, M. Khogali, R. Zachariah, S. Dar Berger, S. Satyanarayana, A. M. V. Kumar, A. F. Bochner, A. McClelland

**Affiliations:** ^1^ Center for Operational Research, International Union Against Tuberculosis and Lung Disease (The Union), Paris, France;; ^2^ Department of Clinical Research, Faculty of Infectious and Tropical Diseases, London School of Hygiene & Tropical Medicine, London, UK;; ^3^ The Union South-East Asia Office, New Delhi,; ^4^ REACH – Resource Group for Education and Advocacy for Community Health, Chennai, India;; ^5^ Respiratory Society of Kenya, Nairobi, Kenya;; ^6^ Department of Medicine, Dermatology and Therapeutics, Kenyatta University School of Medicine, Nairobi, Kenya;; ^7^ Department of Medicine, Aga Khan University, Karachi, Pakistan;; ^8^ Common Management Unit (TB, HIV/AIDS & Malaria), Islamabad, Pakistan;; ^9^ Institute of Public Health, College of Medicine and Health Sciences, University of the United Arab Emirates, Al Ain, UAE;; ^10^ United Nations Children Fund, United Nations Development Programme, World Bank, WHO Special Programme for Research and Training in Tropical Diseases (TDR), WHO, Geneva, Switzerland;; ^11^ Yenepoya Medical College, Yenepoya (deemed University), Mangalore, India;; ^12^ Resolve to Save Lives, New York City, NY, USA.

Timeliness is a crucial concept in medicine and public health, and is defined as ‘a fact or happening occurring at the best possible time or the right time’.^1^ A classic example for infectious diseases is acute meningococcal meningitis, where prompt diagnosis and treatment are essential to prevent severe morbidity and death. Treatment for this dangerous infection is recommended to be started as soon as possible, but at least within one hour of presentation.^2^ For *Neisseria meningitidis,* close contacts are also at high risk of acquiring infection and developing clinical disease, which can be prevented by prompt chemoprophylaxis. Here too, timeliness is essential, as chemoprophylaxis should be started within 24 hours of the index patient being diagnosed to achieve maximum benefit.^3^ Similarly, with a chronic infectious disease such as TB, timeliness is of the essence. Delays in diagnosis and treatment initiation, which are still common in many low- and middle-income countries (LMICs), result in poor individual treatment outcomes, including death.^4,5^ Treatment delays affect the individual but also increase the risk of transmission of *Mycobacterium tuberculosis* in the community. Household contacts (HHCs) are at particular risk.^6,7^ There is a dose-response relationship between delay of treatment in the index patient and infection in HHCs, especially for infants and young children, with 30 days appearing to be the turning point at which a significant increase in risk begins to occur.^8^ Other studies have endorsed these findings, showing that 83% of child HHCs with TB infection develop active disease within 90 days of exposure.^9^

However, timely provision of TB preventive therapy (TPT) can reduce the risk of developing TB disease by 63% among all children exposed to active pulmonary TB patients, and by 91% among children who are identified with TB infection.^9^ Further evidence has led to the WHO recommending TPT for all HHCs, adults and children, after active TB is ruled out,^10^ with the benefit of TPT lasting for several years after completing the course.^11,12^ However, timeliness in initiation of TPT remains sub-optimal, especially in LMICs, and studies and anecdotal reports have shown that HHC screening and initiation of TPT among eligible contacts can take up to 2 months or longer, negating its value in breaking household transmission.^13,14^

To improve timeliness for HHC screening, management and initiation of TPT, we were inspired by a new global target of ‘7-1-7’ proposed to facilitate early detection and rapid control of health threats such as suspected infectious disease outbreaks.^15,16^ This is defined as follows: the detection of an outbreak within 7 days of emergence, the notification of the outbreak to public health authorities within 1 day of detection, and the completion of early response actions within 7 days of notification. We adapted this metric for household contact management as shown in Table 1. We then assessed the feasibility and usefulness of this metric to screen, manage and provide TPT in HHCs of index patients with pulmonary TB in four specific contexts: the private sector through TB *Nanbans* (meaning ‘friends’ in Tamil), in Chennai, India; the public sector through TB survivors in Chhattisgarh, Bihar and Odisha states, India; tertiary health facilities through dedicated project staff, Sindh Province, Pakistan; and through healthcare workers within the National TB Programme, Kiambu County, Kenya. The methodology and data capture/validation processes were similar in all the sites, as described in a series of related papers.^17–19^

**Table 1.** Use of ‘7-1-7’ timeliness metrics in the context of outbreak preparedness and household contact screening and management of pulmonary TB patients (adapted from reference 19).

Timeliness metrics	Outbreak preparedness	Household contact management
First-7	Detection of an outbreak within 7 days of its emergence	Household contacts of the index TB patient are line-listed within 7 days of the index patient initiating anti-TB treatment
Next-1	Notification of the outbreak to public health authorities within 1 day of detection	Line-listed household contacts are screened for symptoms suggestive of TB within the next 1 day of line-listing
Second-7	Completion of early response actions within 7 days of notification	Eligible household contacts are initiated on anti-TB treatment, TB preventive treatment or a decision is taken to not provide/receive drugs within 7 days of symptom screening

The key findings are presented in Table 2. First-7 was the component most easily achieved at 91%. Overall achievement of the Next-1 was 79%, the key challenge here being face-to-face screening of HHCs within 1 day. Children and adult members of the household were frequently away at school or at work, necessitating return visits to the homestead or arranging visits on another day to the health facility or resorting to phone-based symptom screening. Second-7 was the most difficult to achieve, with only 50% of HHCs eligible for further evaluation and action receiving TPT, anti-TB treatment or a decision to not provide or receive drugs. There were three key barriers: 1) reluctance of HHCs to attend and be assessed in the clinics due to travel costs, loss of daily earnings, fear and stigma associated with TB and unwillingness to take medication while they felt well; 2) hesitance of healthcare workers to provide TPT due to lack of clear guidelines and fear of adverse drug reactions and/or development of drug resistance; and 3) shortages of drugs for TPT. In settings where historical data on the TPT cascade were available, 890/1,825 (49%) HHCs were initiated on TPT at any time after screening during the 7-1-7 period compared to 365/1,318 (28%) of HHCs 3 months before the 7-1-7 metric was implemented.

**Table 2.** Implementing ‘7-1-7’ metrics for HHC screening and management in India, Pakistan and Kenya, 2023.

7-1-7 component	Study site^17–19^	Percentage achieved %	7-1-7 component	
First-7			Index TB patients registered whose HHCs were listed within the first 7 days of index patient’s treatment	
			Numerator (index patients completing activity *n*)	Denominator (index patients registered *n*)
	India, private sector	90	237	263
	India, public sector	89	527	595
	Pakistan, Government	92	414	450
	Kenya NTP	95	483	508
	Total	91	1,661	1,816
Next-1			Listed HHCs who had symptom screening outcomes completed within next 1 day	
			Numerator (HHC completing activity *n*)*	Denominator (HHC who were listed *n*)
	India, private sector	48	267	556
	India, public sector	90	1,896	2,108
	Pakistan, Government	84	1,121	1,342
	Kenya NTP	68	790	1,160
	Total	79	4,074	5,166
Second-7			HHCs who started treatment with TPT, ATT or a decision was taken that no drugs would be given within next 7 days	
			Numerator (HHCs for whom decision/action taken *n*)^†‡^	Denominator (HHCs eligible for follow-up evaluation *n*)
	India, private sector	57	259	454
	India, public sector	42	874	2,073
	Pakistan, Government^§^	35	89	256
	Kenya NTP	65	722	1,105
	Total	50	1,944	3,888

* Numerator does not include HHCs who did not consent or HHCs who were screened for symptoms outside of the specified time period.

^†^ Numerator does not include HHCs who started TPT, ATT or were not given any drugs after the 7-day time period.

^‡^ Numerator comprises of number started on TPT = 1,398; number started on ATT = 18; and number for whom a decision was taken not to give any drugs = 528.

^§^ Of those completing symptom screening, only 256 (children aged <5 years and adults with chest symptoms) were eligible for further evaluation and action according to national guidelines.

HHC = household contact; NTP = National TB Programme; TPT = TB preventive therapy; ATT = anti-TB treatment.

In all the sites, the field staff found the timeliness metrics feasible and useful to implement. Field staff in Pakistan strongly recommended that timeliness metrics be integrated within the National TB Programme guidelines to optimise HHC screening and management. The clear steps combined with explicit timelines gave structure to the various tasks needing to be done. Staff found the metrics to be motivating and they were perceived to lead to improved performance. J Mwangi, Department of Health, Kiambu, Kenya, stated that:

‘TPT uptake amongst household contacts increased in sites implementing 7-1-7.’

Field staff in the three countries suggested that an adapted timeliness metric of ‘3-5-7’ (defined as 3 days to line-list HHCs, and 5 days to obtain symptom screening outcomes of HHCs) might be a more workable alternative, as this would provide the time needed to meet all of the HHCs personally and screen them for symptoms of TB. As we captured the dates for when each individual achieved the three components of ‘7-1-7’, we were able to extrapolate what could be achieved with a First-3 and a Next-5 metric. In our total study population, 76% of HHCs could have been line-listed by index patients within 3 days compared with 91% using 7 days. Although a decline in meeting the first target from 91% to 76% is large, had a 3-day target been set from the beginning, we believe that performance would have been better. Completing the symptom screening outcome component improved from 79% in 1 day to 88% in 5 days. Increasing the Second-7 to more days would not solve the current challenges mentioned above, which require HHCs and healthcare providers to have better awareness of the benefits of TPT and strengthened health system/health service structure to support the intervention, as has been recommended by others.^20^

In 2018, world leaders committed to provide TPT to at least 24 million HHCs by 2022.^21^ Implementation, unfortunately, was sub-optimal, with only 4.2 million (17.5% of target) HHCs receiving this intervention in the 5-year period.^22^ Undaunted, at the second United Nations High-Level Meeting on TB in September 2023, world leaders again emphasised the importance of TPT and pledged to deliver TPT to 30 million HHCs by 2027.^23^

Prevention is better than cure. Many patients successfully completing anti-TB treatment suffer from substantial disability,^24^ and have a three-fold higher risk of death in the follow-up period compared with those who have never had TB.^25^ Programmes and their implementing partners need to embrace HHC screening and management and find innovative ways to make TPT work. We believe that applying ‘timeliness’ metrics to the screening and management cascade for HHCs adds structure and a sense of urgency to this intervention, and timeliness should be monitored within the routine system. Whether ‘7-1-7’ is the ideal metric for this activity or the proposed ‘3-5-7’ would work better needs further assessment under operational research conditions. We strongly encourage others to take up the ‘timeliness’ ethic to strengthen the evidence around its feasibility and value.
